# Post-COVID Syndrome and Severity of COVID-19: A Cross-Sectional Epidemiological Evaluation From North India

**DOI:** 10.7759/cureus.27345

**Published:** 2022-07-27

**Authors:** Nidhi Uniyal, Yashendra Sethi, Pradeep C Sharma, Ashutosh Sayana, Narayan Jeet, Anurag Agarwal, Vijay Rawat

**Affiliations:** 1 Department of Medicine, Government Doon Medical College, Dehradun, IND; 2 Department of Surgery, Government Doon Medical College, Dehradun, IND; 3 Department of Tuberculosis and Chest Medicine, Government Doon Medical College, Dehradun, IND; 4 Statistics, Shri Guru Ram Rai Degree College, Dehradun, IND

**Keywords:** long covid-19, acute covid illness, long covid haulers, post covid syndrome, post-acute covid, covid

## Abstract

Background

COVID-19 has now lasted for more than two years as a pandemic and has had enduring effects on the health of people as the post-COVID syndrome. Recent literature has shown the long-term effects of COVID‐19 on various organ systems, including but not limited to respiratory, cardiovascular, neurological, musculoskeletal, and gastrointestinal systems.

Methods and objectives

We aimed to estimate the prevalence of post-acute COVID symptoms in a tertiary care center in northern India; observe the effects of the demographic profile of age, BMI, gender, and presence of comorbidities on the persistence of post-COVID syndrome, and explore any correlation between the severity of COVID-19 disease and the persistence of post-COVID symptoms. We designed a survey containing structured questions evaluating post-COVID symptoms beyond three weeks (post-acute COVID phase), six weeks (post-COVID phase), and 12 weeks of acute illness. It was administered online.

Results

Prevalence of post-COVID symptoms both after three and six weeks was reported to be 16.67% and 7.37%, respectively. The most common symptoms to persist were musculoskeletal symptoms (fatigue), followed by upper respiratory symptoms. Disease severity (p<0.05), BMI (p<0.05), and comorbidities were seen to affect post-COVID symptoms significantly, whereas gender and age of the patient had no significant effect. Disease severity significantly affected the persistence of post-COVID symptoms up to 12 weeks; however, this effect does not hold true in long COVID haulers. Also, the risk of developing persistent post-acute COVID symptoms was more in moderate to severe disease than in mild disease.

Conclusion

The pandemic might be close to over, but it is not out of our lives yet, and the persistence of post-COVID symptoms is exigent.

## Introduction

COVID-19 has been around for more than two years now, and the pandemic has had long-lasting effects on the health of people as a wide spectrum of disease illnesses. It might have reached its nadir but continues to persist subtly as the post-COVID syndrome in people who got infected and were later declared cured.

Patients, even those having mild symptoms of COVID-19, have continued to experience symptoms after initial recovery [1,2]. Post-infectious symptoms have been recognized for some other illnesses as well [3,4]. For example, the severe acute respiratory syndrome (SARS) infection also had patients with residual damage in the lungs [5,6]. Moreover, some studies have described psychological complaints, fatigue, limitations to fitness, and reduced quality of life after a SARS infection [7,8]. Other diseases like Q fever, Legionnaires' disease, glandular fever, and epidemic polyarthritis have also shown post-disease illnesses like fatigue, neurocognitive problems, musculoskeletal pain, and mood disturbance six months after these infections [3,9]. The patients with COVID-19 were therefore always on the verge of facing post-disease illness syndrome.

The existing literature describes a complete spectrum of symptoms affecting different systems of the body leading to neurocognitive post-COVID (brain fog, dizziness, loss of attention, confusion), autonomic post-COVID (chest pain, tachycardia, palpitations), gastrointestinal post-COVID (diarrhea, abdominal pain, vomiting), respiratory post-COVID (general fatigue, dyspnea, cough, throat pain), musculoskeletal post-COVID (myalgias, arthralgias), psychological-related post-COVID (post-traumatic stress disorder, anxiety, depression, insomnia), and other manifestations (ageusia, anosmia, parosmia, skin rashes) [10-15]. Furthermore, thyroid abnormalities seen in the acute phase of COVID-19 [16] have also been reported to persist or reappear during the post-COVID phase [17].

A growing number of studies have reported the disease to be persisting beyond the acute phase for weeks or even months in the form of multiple symptoms varying from fatiguability to cardiac and neurological manifestation. These manifestations exist irrespective of the disease severity in the acute phase.

To date, there are limited data on persistent symptoms in patients after a mild COVID-19 infection (patients managed in an outpatient setting). Most literature encompasses patients with severe infections (patients managed in an inpatient setting) [18]. There is a dearth of data, especially from North India, for mild to moderate patients. Therefore, we aimed not only to add data on patients with mild COVID-19 symptoms but also to observe the prevalence of post-COVID symptoms in this region. The study was done with the prime objective of estimating the prevalence of post-acute COVID symptoms in the population of northern India. Also, to observe the effect of the demographic profile of age, BMI, and gender on the persistence of post-COVID syndrome and to explore any correlation between the severity of COVID-19 disease and the persistence of post-COVID symptoms.

## Materials and methods

The study was conducted at a tertiary health care center in North India, collecting data from a sample of 400 (as estimated by Cochrane's formula) derived from the patients followed up in post-COVID outpatient clinics. Our study criteria for labeling post-acute COVID were based on the Indian Council of Medical Research (ICMR) discharge policy: asymptomatic patients discharged after 10 days of positive reverse transcription-polymerase chain reaction (RT-PCR) test reports and symptomatic patients within 10 days of onset of symptom and no fever for three days with oxygen saturation (SpO_2_) of 94% at room air with positive RT-PCR test reports. The patient was further recommended a period of home isolation of seven days as per the national guidelines. The study was done with the help of an online questionnaire consisting of questions pertaining to the demographic profile of patients, their comorbidities, the clinical presentation at the time of diagnosis, the medications taken by them during the disease, and the presence of symptoms beyond three weeks (post-acute COVID phase), six weeks (post-COVID phase) and 12 weeks.

The following case definitions were used [19]. As the study included the patients at home isolation, we kept the duration of three weeks for acute COVID illness. Symptoms persisting beyond three weeks up to six weeks were defined as post-acute COVID illness. Symptoms persisting beyond six weeks to 12 weeks (three months) were defined as a post-COVID syndrome, and individuals with symptoms persisting beyond 12 weeks (three months) were called long-COVID haulers.

The data was analyzed for both categories of patients: patients with home isolation treatment and patients with hospitalization. The severity of the disease was determined by the stated two categories. Patients in home isolation or those hospitalized but not on oxygen therapy were kept under the mild category, and those hospitalized but needing oxygen therapy (with either a nasal cannula, mask, high flow mask, or ventilation) were categorized as moderate to severe.

We included COVID-19 RT-PCR positive patients who were asymptomatic or had mild disease, hospitalized or non-hospitalized (home isolation) till 15th May 2021, or those with moderate or severe disease who were hospitalized and had SpO_2_ more than 94% at room air and were afebrile for three days before being discharged.

Respondents who denied consent for participation were excluded from the study. We also excluded patients who filled the form incompletely or with incoherent responses or those who, at the time of discharge, did not attain the SpO_2_ of 94% or more than 94% at room air to remove lung sequelae as confounding factors.

The ethical approval was taken from Government Doon Medical College with reference number GDMC/IEC/2022/10. The participants were given all details of the study and were allowed voluntary informed consent through the online questionnaire with full knowledge of the possible risks and health benefits of participation.

The statistical tests were applied using SPSS 27.0 version for Windows (IBM Inc., Armonk, USA). Categorical variables were expressed in percentages. We applied the Pearson Chi-square test to assess the categorical variables, whilst continuous variables were depicted as mean with standard deviation. The data were compared among the two possible groups, the one which had persistent post-COVID symptoms and the other which had no post-COVID symptoms. The independent variables used in the study were age, sex, body mass index, smoking, drinking, comorbidities, presenting symptoms, the severity of disease, and the medications used in the treatment of illness.

## Results

The survey had 406 responses, out of which 387 patients agreed to participate in the study. Only 383 were taken for further analysis as four people filled spurious data. Seventeen respondents were asymptomatic at the time of the RT-PCR test and detected by contact tracing, out of which 16 remained asymptomatic, while one patient developed symptoms later and was hospitalized.

**Figure 1:**
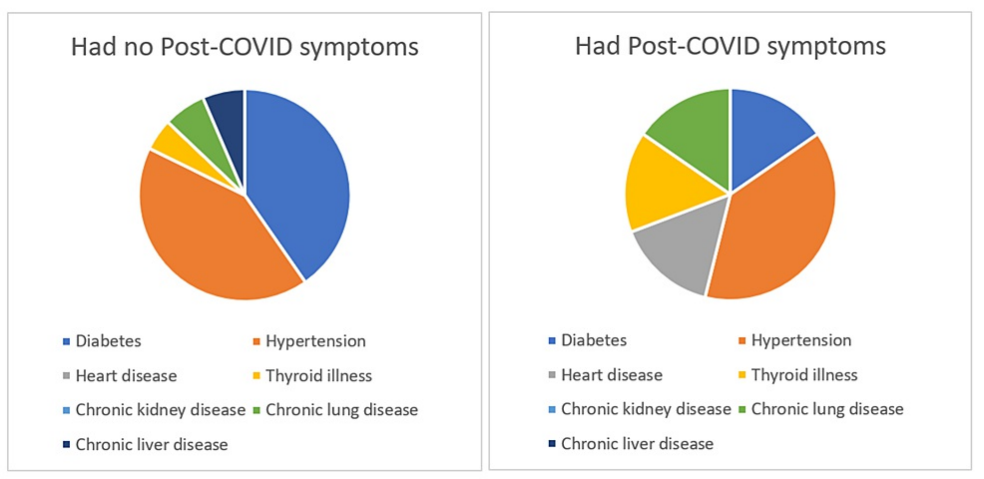
Comorbidities and persistence of post-COVID symptoms

We found that, among the 383 respondents, 83 (21.68%) were hospitalized during treatment, and 300 (78.32%) were in home isolation. Of the 360 patients analyzed for post-acute COVID symptoms (persistence of symptoms beyond three weeks), 23 were excluded as they responded before the completion of their three weeks of illness. Out of 360 people, who qualified for post three weeks symptoms, 110 were with comorbidities, of which 21 developed post-COVID symptoms (19.09%). While 39 out of 250 people with no comorbidity (15.6%) developed the post-COVID symptoms, and the difference was statically significant (p<0.0001). Hypertension, diabetes, and heart disease were the most common comorbidities in our sample. It was seen that the group of patients in the sample who developed post-COVID symptoms had more prevalence of pre-existing heart disease (Figure 1).

**Figure 2:**
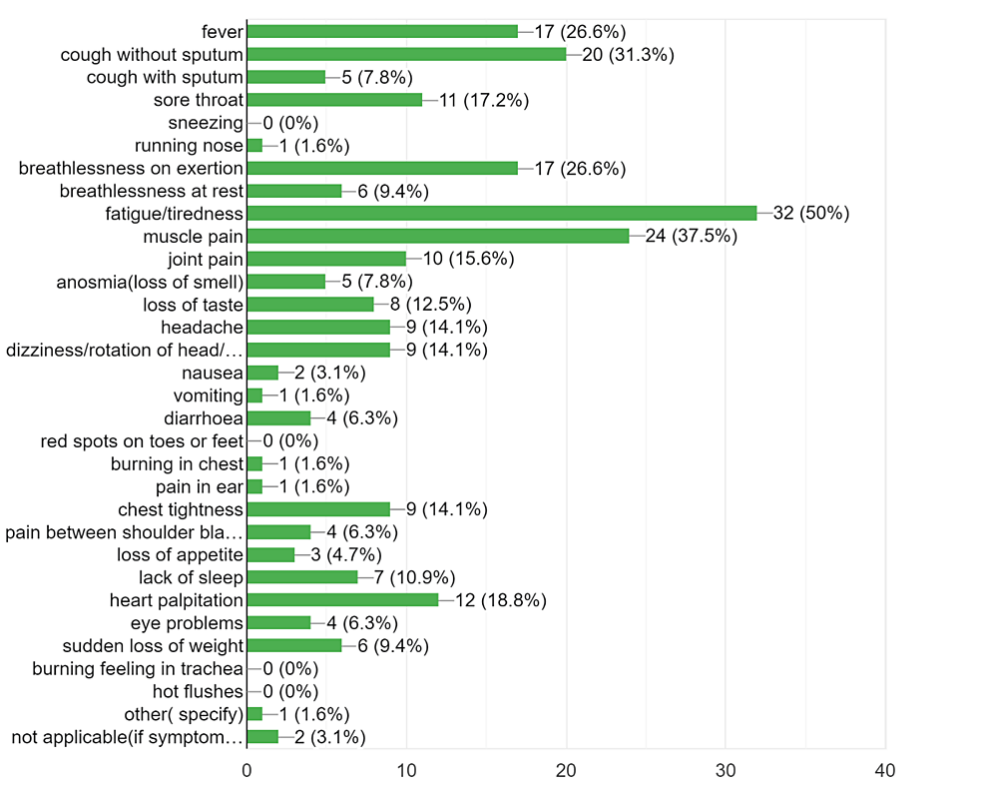
The persistence of post-COVID symptoms from three to six weeks

**Figure 3:**
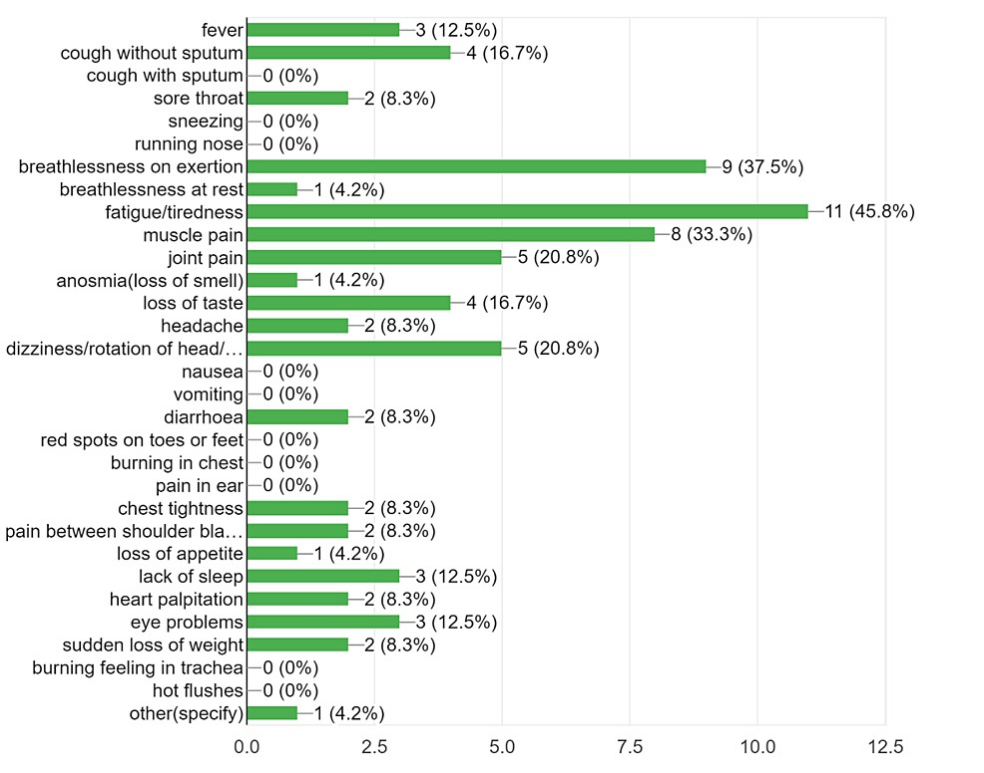
The persistence of post-COVID symptoms from six weeks to three months

**Figure 4:**
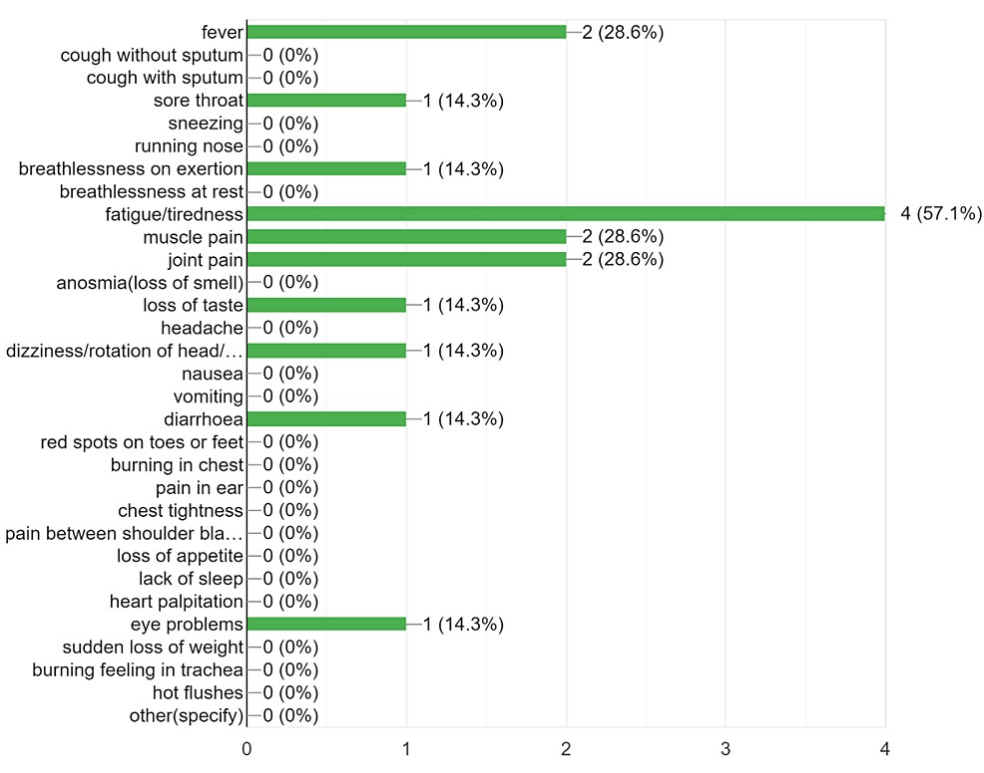
The persistence of post-COVID symptoms beyond three months.

**Figure 5:**
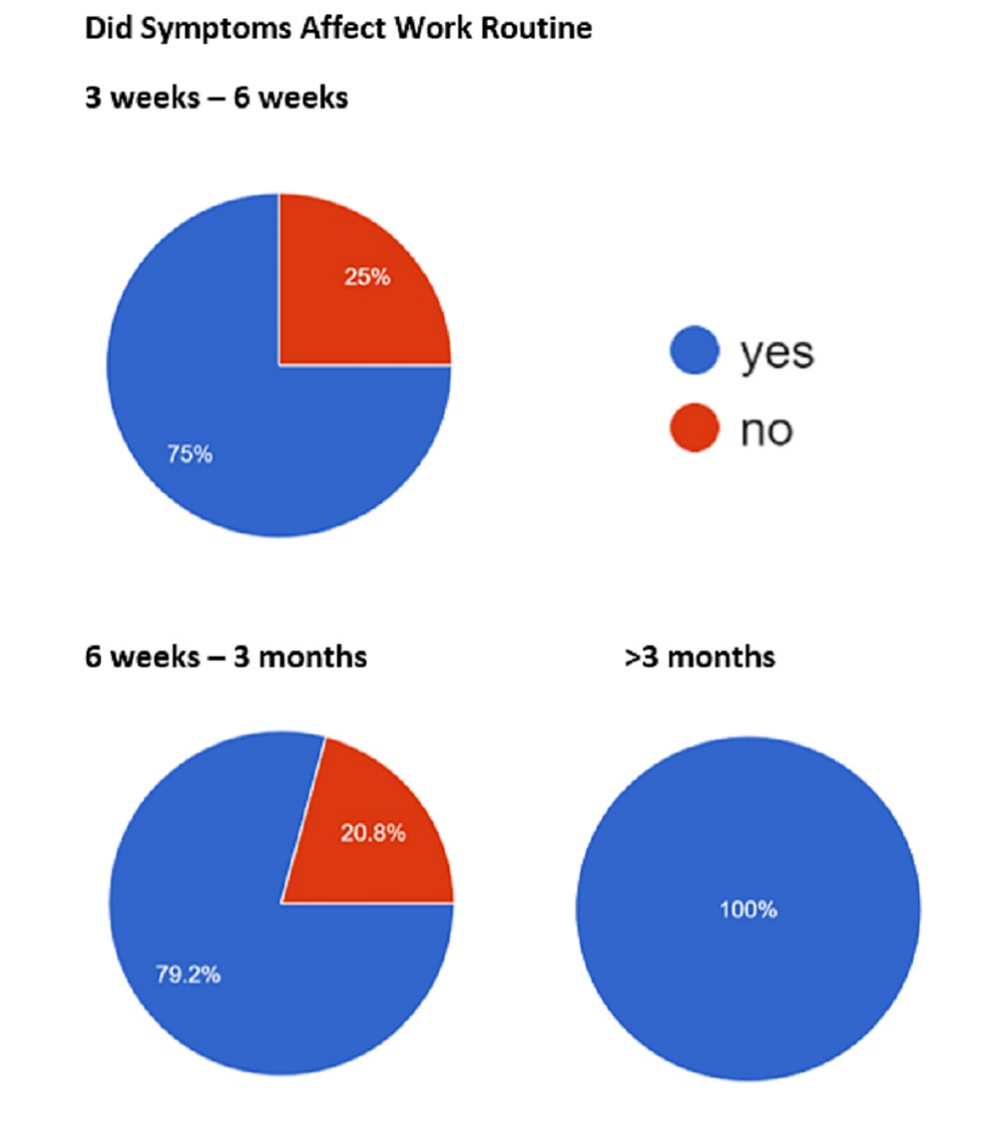
The effect of post-COVID symptoms on work routine

The prevalence of post-acute COVID symptoms in our study was 16.67%, while beyond six weeks, the prevalence rate of post-COVID symptoms persistence was 7.38% (Table 1). We found that the most common symptoms to persist in the post-acute COVID and post-COVID phase were the musculoskeletal symptoms in the form of fatigue, persistent myalgia, and tiredness, followed by upper respiratory symptoms, breathlessness, fever, headache, and cognitive/psychosomatic symptoms (Figures 2-4). Furthermore, the patients also reported that the duration of persistence of symptoms directly affected the daily work routine (Figure 5).

**Table 1:** Percentage distribution of various post-COVID symptoms URT - upper respiratory tract

	At the time of diagnosis		Post three weeks		Post six weeks	
Category of symptoms	Frequency	Percentage	Frequency	Percentage	Frequency	Percentage
Fever	338	88.25%	16	26.67%	3	16.67%
Musculoskeletal	258	67.84%	38	63.33%	12	66.67%
URT symptoms	201	52.48%	29	48.33%	4	22.22%
Headache/giddiness	105	27.42%	13	21.67%	6	33.33%
Cognitive/psychosomatic	57	14.88%	5	8.33%	2	11.11%
Gastrointestinal	89	23.23%	9	15%	1	5.56%
Loss of taste/smell	111	28.98%	9	15%	0	0
Breathlessness	61	15.92%	21	35%	7	38.89%

It was seen in our study that a higher fraction of patients with moderate to severe disease had persistent post-COVID symptoms for six and 12 weeks as compared to patients with mild disease (Table 2).

**Table 2:** Post-COVID syndrome as per the severity of the disease Results were considered significant at p <0.05.

	Post three weeks			Post six weeks			Post 12 weeks		
	(n=360)			(n=244)			(n=123)		
Post-COVID symptoms	Present	Absent	Total	Present	Absent	Total	Present	Absent	Total
Mild	46	278	324	12	205	217	2	110	112
Moderate to severe	14	22	36	6	21	27	1	10	11
Total	60	300	360	18	226	244	3	120	123
X^2^	14.2222 (p=0.0002)			9.7918 (p=0.0018)			2.2463 (p=0.1339)		

The demographic analysis of data suggested interesting differences between the patients who developed post-COVID symptoms vs. those who did not develop them. The data suggested that age, BMI, and duration of hospitalization played a significant role (Table 3).

**Table 3:** Comparison of demographic profiles of patients who had persistent post-COVID symptoms and the patients who did not have any persistent post-COVID symptoms Significant at p<0.05.

Variable	Post three weeks (n=360)		Post six weeks (n=244)	
	With post-COVID symptoms	Without post-COVID symptoms	With post-COVID symptoms	Without post-COVID symptoms
Age (mean/SD)	41.07 +/-15.78	42.69 +/-13.97	43.94 +/-11.37	43.32 +/-12.85
X^2^	0.2066 (p=0.901)		2.4712 (p=0.290)	
BMI (mean/SD)	26.75 +/-5.06	25.85 +/-4.57	28.63 +/-4.19	26.11 +/-4.64
X^2^	0.3975 (p=0.173)		0.29 (p=0.026)	
Gender				
Male	38	194	11	162
Female	22	106	7	64
X^2^	0.0388 (p=0.843)		0.9029 (p=0.342)	
Smoker				
Yes	n=22		n=18	
	3	19	1	17
No	n=338		n=226	
	57	281	17	209
X^2^	0.1549 (p=0.693)		0.0944 (p=0.759)	
Alcoholic				
Yes	n=48		n=40	
	7	41	2	38
No	n=312		n=204	
	53	259	16	188
X^2^	0.1731 (p=0.677)		0.3956 (p=0.529)	
Age groups	n=360		n=244	
14 - 39 years	23	118	4	82
40 - 59 years	31	147	13	120
≥60 years	6	35	1	24
X^2^	0.2066 (p=0.901)		2.4712 (p=0.290)	
Home isolation	n=280		n=174	
With medication	38	212	12	137
Without medication	1	29	0	25
Hospitalization	n=23		n=20	
≤7 days	2	9	0	10
8 - 14 days	1	6	0	7
>14 days	4	1	2	1
X^2^	7.441 (p=0.024)			
Comorbidities	n=360			
With comorbidities	21	89		
Without comorbidities	39	211		

The persistence of post-COVID symptoms and the effect of medications were studied only for the sample in home isolation as they constituted the sample with mild cases which had a limited number of medications, allowing easy recall to report in our questionnaire. Also, the hospitalized patients had more medications given by hospital staff, hence having lesser chances of recall by the patients. Out of 280 patients in home isolation, 221 patients took ivermectin or hydroxychloroquine (HCQS) or both as a treatment, and of these, 33 (14.9%) had persisting post-COVID symptoms (Table 4).

**Table 4:** Distribution of ivermectin and HCQS intake by home isolation group HCQS - hydroxychloroquine

	HCQS	Steroids	Ivermectin	HCQS + steroid	Ivermectin + steroid	HCQS + ivermectin without steroid	HCQS + ivermectin with steroid	Others or not known
n=280	4	11	116	3	60	28	10	48
Post-COVID	0	0	18	0	9	3	3	5

## Discussion

In our study, the most common symptoms which persist in the post-acute COVID and post-COVID phase were the musculoskeletal symptoms in the form of fatigue and persistent myalgia, followed by upper respiratory symptoms (sore throat, cough), breathlessness, fever, headache, and cognitive/psychosomatic symptoms in the form of lack of sleep, lack of attention (Table 1). Contextually Lopez-Leon et al. reported 'fatigue' as the commonest symptoms to manifest post-COVID syndrome, followed by headache, attention disorder, hair loss, and dyspnoea [20].

The sociodemographic features were similar to the study by Mahmud et al. [21]. Our study had approximately 40% of people aged less than 40 years and around 11% aged more than 60 years, as compared to 60% and 8%, respectively, in the referred study.No significant difference was found between the age profile of patients among the post-COVID symptoms groups and the non-post-COVID symptoms group, which indicates that all age groups were equally susceptible to developing post-COVID syndrome [21,22].

In our study, no significant difference was observed between males and females in developing the post-COVID syndrome. Interestingly this is in contrast to the results reported by Mahmud et al. [21] in their study, which showed male gender preponderance in the persistence of post-COVID symptoms. Sex ratio in our study was 1.8:1 (n[M:F] =232:128, n=360 vs. n1[M: F] =207:148, n1=355; 95% CI, p>0.05, not significant) comparable to 1.4:1 in the referred study.

We analyzed the effect of smoking and alcohol intake on the persistence of post-COVID symptoms and found no significant difference in the incidence of post-COVID syndrome in either of the categories compared to controls (Table 3). People with higher BMI were more vulnerable to developing persistence of post-COVID symptoms post six weeks (mean=28.63 +/-4.19 vs. 26.11 +/-4.64; 95% CI: 0.29-4.7, p=0.026) compared to those with lower BMI. This is in accordance with a previous study by Klaser et al., who found stronger associations of unhealthy BMI categories with anxiety/depression symptoms: ORs of 1.26 (95% CI: 1.22-1.30, p<0.001), 1.21 (95% CI: 1.20-1.22, p<0.001) and 1.61 (95% CI: 1.59-1.62, p<0.001), respectively [22].

Coexisting comorbidities were observed as an independent risk factor for the persistence of post-COVID symptoms (Figure 1), which were similar to a previous study by Gala et al. [23]. The severity of the disease was associated with the development of post-COVID syndrome (Table 2). The risk of developing post-acute COVID symptoms beyond three weeks in mild COVID-19 illness was 14%, while that in the moderate to severe group was 39%. This risk remains at 6% in mild disease and 29% in moderate to severe group beyond six weeks post-COVID. Our study reported that the disease severity does significantly affect the persistence of post-COVID symptoms up to 12 weeks, but this effect does not hold true beyond 12 weeks. Mahmud et al. [21] made a similar observation in their study. In hospitalized patients, the number of days of hospitalization was significantly associated with the persistence of symptoms three weeks post-COVID (p=0.024). None of the patients hospitalized for less than 14 days were reported to have post-COVID symptoms beyond six weeks in our study, indicating that the long COVID syndrome was independent not only of the disease severity at the time of presentation but also during the period of hospitalization, once the patient is discharged after full recovery [24].

In our study, the prevalence of post-COVID symptoms both after three and six weeks was reported to be 16.67% and 7.37%, respectively, compared to the 47%-60% prevalence reported in other studies [20,23]. The study included the individuals who were declared recovered as per the Indian government guidelines, according to which those who were able to maintain oxygen saturation of more than 94% at room air and were afebrile for three consecutive days were declared recovered. The exclusion of the individuals from the study with more than 21 days of hospitalization and individuals discharged on prolonged oxygen therapy might be an explainable cause for the low prevalence of post-COVID symptoms compared to other studies, which included all patients irrespective of their recovery status. A study by Mahmud et al. [21] excluded patients admitted to the critical care unit to avoid the patients with the risk of developing post-intensive care sequalae/stress and reported a prevalence of around 46% (162/355 vs. 60/360, p<0.00001), which confirms the possible association of early treatment by ivermectin or hydroxychloroquine may have a beneficial effect on persistence of post-COVID symptoms and this may be attributed to the possible effect of ivermectin (as treatment or prophylaxis) on disease severity [25,26]. This study enrolled around 79% of patients in home isolation who had taken either/both of the two medicines (Table 4). Ivermectin and hydroxychloroquine both have immunomodulatory and anti-inflammatory effects. The commonest post-COVID symptom is fatigue, as reported in various studies, including this study, being compared to chronic fatigue syndrome. Raised interleukin 6 (IL-6) was found to be associated with chronic fatigue syndrome [27]. Thus indicating the possible association of raised inflammatory cytokines and persistence of post-COVID symptoms, which may have been countered by prophylaxis given to the patients.

Limitations

The study limitation is that responses are subjective based on the replies to the questionnaire and thereby depend on the interpretation of the responder and are not supported by any objective assessment of symptoms. Secondly, it is a single-center study and, thus, lacks a diverse sample, limiting the generalizability of our findings. Lastly, the majority of patients enrolled for the study received treatment irrespective of disease manifestation as per the state treatment guidelines and thus cannot rule out the bias in reporting the low prevalence of post-COVID symptoms in our study sample compared to other studies.

## Conclusions

The purpose of this study was to observe the prevalence of post-COVID symptoms in the region of northern India to find any effect on the prevalence of post-COVID symptoms due to treatment of the mild/asymptomatic group. Noticeably, we found that the most common symptoms which persist in the post-acute COVID and post-COVID phase were the musculoskeletal symptoms (fatigue), followed by upper respiratory symptoms. No significant difference was found in different age groups or genders, though unhealthy BMI was seen to be a statistically significant (p<0.05) factor. Comorbidities were observed as an independent risk factor. Disease severity did significantly affect the persistence of post-COVID symptoms up to 12 weeks; however, this effect does not hold true in long COVID haulers. The risk of developing and persistence of post-acute COVID symptoms was clearly found to be more in moderate disease severity than the mild disease. Early treatment by ivermectin or hydroxychloroquine may be a probable cause for the low prevalence of post-COVID symptoms in the study, though we need more exploration on this aspect. Future studies are warranted to observe the long-term effects of COVID-19 and also explore the post-COVID effects using multicentric studies. The pandemic might be close to over, but it is not out of our lives yet.
